# The Impact of Life Trauma on Mental Health and Suicidal Behavior: A Study from Portuguese Language Countries

**DOI:** 10.3390/bs12040102

**Published:** 2022-04-10

**Authors:** Mariana Silva, Henrique Pereira

**Affiliations:** 1 Department of Psychology and Education, Faculty of Social and Human Sciences, University of Beira Interior, Pólo IV, 6200-209 Covilhã, Portugal; mariana.t.silva@ubi.pt; 2 Research Centre in Sports Sciences, Health Sciences and Human Development (CIDESD), 5001-801 Vila Real, Portugal

**Keywords:** life trauma, traumatic experiences, mental health, suicidal behavior, suicidal thoughts, suicide attempts, Community of Portuguese Language Countries

## Abstract

Several studies report the incidence of traumatic experiences in community and clinical samples, and substantial research demonstrates the impact of traumatic events on mental health and suicidal behavior, but this area remains unexplored in the Community of Portuguese Language Countries (CPLC). Thus, this study aims to (1) describe traumatic experiences, mental health levels and suicidal behavior among individuals from Portugal, Brazil and African Countries with Portuguese as an Official Language (ACPOL); (2) assess correlations between traumatic experiences and mental health and suicidal behavior; and (3) assess the impact of exposure to a traumatic event on mental health and suicidal behavior. The measurement instruments included a sociodemographic questionnaire, Brief Trauma Questionnaire, Brief Symptoms Inventory-18, and the Portuguese version of the Suicidal Behaviors Questionnaire-Revised. ACPOL participants reported greater impact of war, Portuguese participants reported greater impact of disasters, and Brazilian participants reported greater impact of psychological and sexual abuse, assault, and death of a family member. Brazilian participants showed the worst levels of mental health and suicidal thoughts. Strong correlations were found between traumatic experiences and mental health levels and suicidal behavior. Traumatic experiences contributed to the explanation of mental health levels and probability of committing suicide.

## 1. Introduction

### 1.1. Traumatic Experiences

The word “trauma” is commonly used to refer to a stressful event; however, each person’s individual adaptability and coping capacity is what defines whether an event is traumatic for them. Because of this, the same event may be traumatic for one person and not for another. When a situation considered traumatic exceeds a person’s coping abilities, it creates psychological trauma [1]. Several studies have reported high rates of different traumatic experiences, and the impact that trauma has on people’s physical and mental health, causing suicidal behavior. However, this issue is still not properly explored in the Community of Portuguese Language Countries.

The World Health Organization (WHO) points out that 40 million children under the age of 15 become victims of violence each year [2]. Recent data report that 3 out of 4 children between the ages of 2 and 4 years suffer from physical and/or psychological violence at home, 1 out of 5 women and 1 out of 13 men are sexually abused before the age of 20 years, and about 120 million girls and young women suffer from some type of forced sexual contact before turning 20 years old [3].

The psychological trauma resulting from a traumatic event depends on the severity of the violence and the person’s experience [2], and can vary from anxiety symptoms and fear to aggressive symptoms and externalizing anger [4], compromising both the mental and physical health of the traumatized individual [2,3].

Several studies report the incidence of exposure to traumatic events in community samples [5,6,7,8,9], clinical samples [10,11,12,13,14,15,16], and specific samples [17,18,19,20,21,22,23]. Several authors report rates of traumatic experiences higher than 50% in the general population [5,21], in clinical populations, such as schizophrenic patients [15] or patients with anxiety and depression disorders [16], and in specific populations, such as institutionalized adolescents [22].

In general, the most reported traumatic experiences are emotional neglect [8,13,16,17,22,24], physical neglect [8,17,18,21,24], and the unexpected death of someone close [5,7,9,21].

Several studies have shown the impact of exposure to traumatic events on mental health, namely anxiety and depression disorders, which are the two main diagnostic categories [25]. Li and collaborators [13] found significant positive correlations between exposure to a traumatic event and anxiety levels in schizophrenic patients, and specifically found that emotional neglect and sexual abuse contribute to a higher probability of anxiety symptoms. In the study by Humphreys and collaborators [26], associations were found between child maltreatment and depressive symptoms, with the strongest correlations among those who experienced emotional abuse and neglect. Van Assche and collaborators [8] found that any childhood trauma was positively correlated with anxiety and/or depression levels, and emotional neglect and abuse were positively related with both anxiety and depressive levels. Other studies have demonstrated similar results with regard to the impact of exposure to a traumatic event on anxiety and/or depressive levels [12,22,24,27,28,29,30,31]. Another widely documented correlation is between exposure to traumatic events and suicidal behavior.

### 1.2. Suicide

Globally, more than 700,000 people die from suicide every year. A 2014 study by O’Hare and collaborators [21] found that 52.1% of participants tried to commit suicide at least once. Furthermore, a study by Hadland and collaborators [32] found that 36.8% of participants reported a history of suicidal ideation, and a study by Capuzzi and collaborators [18] found that 23.7% of participants have a history of suicide attempts.

Suicide is one of the most common causes of death, so it is extremely important to comprehend risk factors of suicide in order to have a complete and directed response for its prevention [33]. Many studies have found that exposure to a traumatic event increases the risk of suicidal behavior [6,10,13,18,21,32,34,35,36,37]. Saraçli and collaborators [24] found that the prevalence of suicidal thoughts among participants who were exposed to some traumatic event (18.3%) was nearly double that of participants without history of trauma during their childhood (9.6%). Mostly, emotional neglect or abuse and physical abuse were found to be linked with higher rates of suicidal behavior [22,38,39].

### 1.3. CPLC

The Community of Portuguese Language Countries (CPLC), an organization founded on 17 July 1996, comprises nine member states: Portugal, Brazil, Angola, Mozambique, Cape Verde, São Tomé and Príncipe, Guinea Bissau, Equatorial Guinea, and East Timor. CPLC occupies an area of about 10.7 million square kilometers across four different continents, and has a population of nearly 250 million. It has, as general objectives, the promotion and diffusion of the Portuguese language, cooperation on several domains, and political–diplomatic concentration between its members. Some of the principles that rule de CPLC are the sovereign equality of its member states, promotion of development and mutually beneficial cooperation, and respect for national identity and territorial integrity (https://www.cplp.org; accessed on 4 February 2022).

The countries in this community share the Portuguese language and some cultural and religious issues, as well as cooperative relations at the economic, medical, and military levels. However, they have their differences in other cultural and religious aspects, as well as in the evolution of each country, namely in the economic sector, security, and daily violence. Therefore, these countries can be considered as a whole due to their similarities, but special attention must be paid to the differences between each one of them.

Given the lack of research on the impact of exposure to a traumatic event on mental health and suicidal behavior in CPLC, the present study aims to (1) describe traumatic experiences, mental health levels, and suicidal behavior, comparing differences between country of residence; (2) assess correlations between traumatic experiences and mental health and suicidal behavior; (3) and assess the impact of exposure to a traumatic event on mental health and suicidal behavior.

Hence, we intend to explore the issues mentioned above in a population with such specific characteristics, making known the difference in how each situation is experienced and its impact on mental health and suicidal behavior. In addition, it is our goal to reinforce the importance of the subject under study and sensitize readers to the issues mentioned throughout the article.

## 2. Materials and Methods

### 2.1. Participants

The study sample consists of 1006 participants, of which 296 (29.4%) live in Portugal, 409 (40.75%) live in Brazil, and 301 (29.9%) live in ACPOL (African Countries with Portuguese as Official Language). The sample is composed of 424 men (41.2%), 576 women (57.3%), and 6 (0.6%) individuals of other genders. The ages range between 18 and 80 years, with a mean age of 41.76 (SD = 14.185). Additional socioeconomic information can be seen in more detail in Table 1.

### 2.2. Measurement Instruments

Participant sociodemographic information was obtained using a questionnaire in which participants indicated their country of residence, age, sexual orientation, ethnicity/race, professional status, educational attainment, socioeconomic status, place of residence, marital status, housing circumstances, and religion.

Participant psychosymptomatology was evaluated using three subscales (somatization, anxiety, and depression) of the Brief Symptom Inventory 18 (BSI-18). These subscales are evaluated by 18 items with Likert-type response scale ranging from 0 = nothing to 4 = extremely. The depression subscale assesses symptoms of depressive disorders such as dysphoric mood states and anhedonia. The anxiety subscale assesses symptoms that indicate panic states, such as nervousness, tension, and motor agitation. The somatic subscale assesses the distress associated with manifestations of automatically regulated systems. The global severity index, obtained through the sum of the 18 items, provides a measurement of the individuals’ general psychological distress levels, where higher scores indicate more intense psychosymptomatology [40]. Cronbach’s alpha, which measures internal reliability for the anxiety, depression and somatic subscales, was 0.87, 0.86 and 0.78, respectively. Cronbach’s alpha for the total scale was 0.93.

An adapted version of the Brief Trauma Questionnaire (BTQ) was used to assess self-reported trauma. While the original version of the BTQ includes 10 dichotomous items [41], this adapted version measures the intensity of the impact of each traumatic experience using a Likert-type response scale ranging from 0 = ‘It didn’t happen to me’ to 9 = ‘It happened and it was very traumatizing’. In the scoring, trauma intensity levels were divided into four categories: 0 = absence (answers 0 on the scale), 1 = mild intensity (answers from 1 to 3 on the scale), 2 = moderate intensity (answers from 4 to 6 on the scale) and 3 = severe intensity (answers from 7 to 9 on the scale). The traumatic experiences reported were: (1) war zone or military area with war tragedies; (2) serious accident; (3) major natural or technological disaster; (4) life-threatening illness; (5) physical maltreatment before age 18; (6) psychological/emotional maltreatment before age 18; (7) sexual maltreatment before age 18; (8) assault, abduction or attack; (9) violent death of a family member or someone close; and (10) witness to death or tragic accident.

To assess the frequency and severity of present and past suicidal behavior, the participant also responded to the Suicidal Behaviors Questionnaire-Revised (SBQ-R) [42]. This study utilized the Portuguese version of this instrument [43], which is composed of four items on a Likert-type response scale that reports lifetime suicidal thoughts (SBQ1), lifetime suicidal attempts (SBQ2), suicidal thoughts in the last year (SBQ3), and suicidal attempts in the last year (SBQ4). The answers to the SBQ1 item ranged from ‘Never’ to ‘I had a plan to commit suicide, and I really wanted to die’; the answers to the SBQ2 item ranged from ‘Never’ to ‘I tried to commit suicide, and I really wanted to die’; the answers to the SBQ3 and SBQ4 items ranged from ‘Never’ to ‘Many times (five or more times)’. The coefficient alpha value for the 4-item SBQ-R scale scores was 0.77. Corrected item total correlations for the 4 items were 0.60, 0.58, 0.54, and 0.55, respectively. A fifth item that asked about the self-assessed probability of committing suicide (SBQ5) and had the same response type, on a scale from 1 = not likely to 7 = very likely, was also included in the measurement of suicidal behavior.

### 2.3. Procedure

The present study was approved by the researchers’ university ethics committee (CE-UBI-Pj-2021-047), and all the objectives were carefully considered before a secure online website was created on Office Forms for research purposes. After approval, the form was disseminated online between May 2021 and mid-October 2021 using both social networks and mailing lists. The participation was voluntary and followed all ethical and deontological principles, and the inclusion criteria were 18 years old or older, connection to any country in the Community of Portuguese Countries, and internet access to answer the questionnaire. Following data collection, all participants’ submitted online data were entered into IBM^®^ SPSS^®^ STATISTICS (version 25). All data cleaning and statistical analysis were performed through this statistical program, using several procedures necessary to fulfill the aims of this study. That is, descriptive statistics were conducted to describe the sample. Student t-tests and ANOVA were performed to evaluate differences between groups. Pearson correlation coefficients were conducted to assess the correlation between traumatic experiences, mental health, and suicidal behavior. And finally, a hierarchical linear regression analysis was performed to explore the effects of independent variables (age, gender, sexual orientation, country of residence, and 10 traumatic experiences) on the dependent variable (mental health and probability to commit suicide).

## 3. Results

The results show that 10.6% of the participants served in a war zone or were in a military area with war tragedies, 27.3% were involved in a serious accident, 13.3% were involved in a major natural or technological disaster, 16.7% had life-threatening illness, 23.8% were physically abused by someone close during childhood or adolescence, 38.9% were psychologically or emotionally abused by someone close during childhood or adolescence, 15.3% were sexually abused by someone close during childhood or adolescence, 43.4% were assaulted, abducted, or attacked, 31% lived through the violent death of a family member or someone close, and 32.3% witnessed death in a tragic accident. Table S1 of Supplemental shows these results in more detail.

When comparing differences in traumatic experiences by country of residence, statistically significant differences (*p* < 0.05) were observed for war [X^2^(6) = 52.418, *p* < 0.001], disasters [X^2^(6) = 16.220, *p* = 0.013], psychological abuse [X^2^(6) = 24.059, *p* = 0.001], sexual abuse [X^2^(6) = 28.952, *p* < 0.001], assault [X^2^(6) = 85.256, *p* < 0.001], and death of a family member [X^2^(6) = 14.032, *p* = 0.029]. As shown in Table 2, Brazilian participants reported the greatest impact from all these traumatic experiences, except war, where ACPOL participants reported the greatest impact, and disaster, where Portuguese participants reported the greatest impact. 

The BSI-18 subscales results show that 43.1% of the participants had anxious symptomatology, 51.3% had depressive symptomatology, and 23.1% had somatic symptomatology. More details about the BSI-18 subscales results are shown in Table S2 of Supplemental. Further analysis examined the differences in mental health levels by county of residence. The results presented in Table 3 show statistically significant differences (*p* < 0.05) in anxiety (F(2; 974) = 17.688; *p* < 0.001), depression (fF2; 977) = 9.716; *p* < 0.001), somatization levels (F(2; 988) = 4.543; *p* = 0.011), and BSI-18 total score (t(2) = 9.425); *p* < 0.001), indicating that participants from Brazil have the worst mental health levels, with higher scores on BSI-18 total scale and all the subscales.

Tukey’s HSD Test for multiple comparisons found that the mean value of somatization was significantly different between Portugal and Brazil (*p* = 0.026, 95% C.I. = [−0.236, −0.012]) and between Brazil and ACPOL (*p* = 0.036, 95% C.I. = [0.006, 0.229]); the mean value of depression was significantly different between Portugal and Brazil (*p* = 0.003, 95% C.I. = [−0.336, −0.056]) and between Brazil and ACPOL (*p* = 0.000, 95% C.I. = [0.101, 0.381]); ]); the mean value of anxiety was significantly different between Portugal and Brazil (*p* = 0.002, 95% C.I. = [−0.341, −0.064]) and between Brazil and ACPOL (*p* = 0.000, 95% C.I. = [0.207, 0.485]); and the mean value of BSI-18 total scale was significantly different between Portugal and Brazil (*p* = 0.002, 95% C.I. = [−0.287, −0.052]) and between Brazil and ACPOL (*p* = 0.000, 95% C.I. = [0.102, 0.335]).

Regarding lifetime suicidal thoughts, 40% of the participants reported a lifetime history of suicidal thoughts and 19.5% reported suicidal thoughts within the last year. In regard to suicide attempts, 10.1% of the participants reported a lifetime suicide attempt and 3.2% of participants reported a suicide attempt in the past year. Furthermore, 19.2% of the participants reported some self-assessed probability of attempting suicide, of which, on a 1–7 scale, 4% reported a self-assessed probability equal to or greater than 4. Table S3 of Supplemental shows all these results in more detail.

Table 4 shows the differences found in suicidal behavior by country of residence. Statistically significant differences (*p* < 0.05) were observed for the SBQ1 (F(2; 945) = 11.441; *p* < 0.001), SBQ3 (F(2;987) = 7.112; *p* = 0.001) and SBQ5 variables (F(2; 981) = 5.525; *p* = 0.004). Brazilian participants were found to have greater lifetime suicidal thoughts and suicidal thoughts in the last year, while Portuguese participants had greater self-assessed probability of committing suicide.

Tukey’s HSD Test for multiple comparisons found that the mean value of SBQ1 was significantly different between Portugal and ACPOL (*p* = 0.010, 95% C.I. = [−0.270, −0.030]) and between Brazil and ACPOL (*p* = 0.000, 95% C.I. = [−0.338, −0.115]); the mean value of SBQ3 was significantly different between Brazil and ACPOL (*p* = 0.001, 95% C.I. = [0.090, 0.386]); and the mean value of SBQ5 was significantly different between Portugal and ACPOL (*p* = 0.005, 95% C.I. = [−0.415, −0.062]) and between Brazil and ACPOL (*p* = 0.029, 95% C.I. = [−0.342, −0.015]).

Using all the variables, two matrix correlations were made to assess the association between the 10 traumatic experiences, mental health levels, and suicidal behavior. Table S4 of Supplemental shows the significant correlations found between anxiety, depression and somatization levels and the different traumatic experiences, and Table S5 of Supplemental shows the significant correlations found between the traumatic experiences and SBQ subscales. Trauma 1 (war) and 3 (disasters) didn’t show significant correlations with any BSI-18 or SBQ-R subscales. Trauma 6 (psychological abuse) showed significant correlations with all BSI-18 and SBQ-R subscales, except for the self-assessed probability of committing suicide. Only trauma 5 (physical abuse) and 7 (sexual abuse) showed significant correlations with all BSI-18 and SBQ-R subscales.

Finally, two multiple linear regressions were created to assess the predictive value of traumatic experiences in mental health levels and in the self-assessed probability of committing suicide. The age, gender, sexual orientation, and socioeconomic status variables were included in the first model; the country of residence variable was added in the second model, and all 10 traumatic experiences were added in the third model. Table 5 shows that all three models were found to be significant, and indicates that traumatic experiences explain 23.4% of the variance in mental health levels, with greater contribution from war, psychological abuse, and witness to death. Similarly, traumatic experiences explain 11.6% of the variance in the self-assessed probability of committing suicide, with greater contribution from accident and psychological abuse, as shown in Table 6.

## 4. Discussion

The purpose of this study was to increase the body of knowledge and scientific literature on trauma, mental health, and suicidal behavior in the Community of Portuguese Language Countries. We described differences in the three variables under study between country of residence, evaluated correlations between exposure to traumatic events and mental health and suicidal behavior, and analyzed the impact that trauma has on both mental health and the self-assessed probability to commit suicide.

Overall, the participants reported a mean of 0.96 for anxious symptomatology, 0.91 for depressive symptomatology, and 0.56 for somatic symptomatology. These values are within the averages for a Portuguese community population [41]. It should be noted, however, that there are no reference values for Brazilian or ACPOL populations.

With regard to suicidal behavior, although the rates of suicidal thoughts and suicide attempts reported in this study are lower than the rates reported by other studies [18,21,33], the self-assessed probability of the participants in the present study committing suicide is higher than that reported by Souza and collaborators [39].

From among the 10 traumatic experiences assessed in this study, assault, psychological abuse, violent death of a family member, and witness to death or tragic accident were the most reported by the participants. This study results are in line with the results of the study of Pham and collaborators [22] and Schmahl and collaborators [16], where emotional/psychological abuse/negligence were traumatic experiences most reported by participants. In the studies of Grasso and collaborators [5] and Wamser-Nanney and collaborators [9], the death of a family member or someone close was one of the traumatic experiences most reported, which corroborates the results of this study. In the revised literature, having been assaulted, abducted, or attacked, and having witnessed a death or a tragic accident, were not reported as common traumatic experiences, but in this study, these are two of the most reported traumatic experiences. This discrepancy can be explained by the differences between the samples, considering that a sample of this study has participants living in countries that often experience conflicts and acts of violence [44].

Although several correlations were found between all traumatic experience under study and the BSI-18 and SBQ-R subscales, only sexual and physical abuse showed significant correlation with all SBQ-R and BSI-18 subscales. The study of Saraçli and collaborators [24] reports a correlation between anxiety levels and exposure to a traumatic event. On the other hand, the study of Huh and collaborators [28] reports a correlation between depression levels and exposure to a traumatic event. The study of Van Assche and collaborators [8] reports a specific correlation between emotional neglect or abuse and both anxiety and depression levels. Additionally, Haug and collaborators [12] reported a correlation between emotional neglect or abuse and suicidal thoughts, and between physical abuse and suicide attempt. All these results corroborate the results of the present study, which shows a correlation between psychological maltreatment and both suicidal thought and attempt and all BSI-18 subscales.

The results of this study show that traumatic experiences contribute to the explanation of mental health levels, namely war, psychological abuse, and witness to death. This adds to the results of the study by Pham and collaborators [22], where emotional abuse and negligence were found to contribute depressive symptoms, and the study of Mal-Sarkar and collaborators [30], where childhood trauma significantly predicted increased depression symptoms. Furthermore, the present study presents psychological abuse as a predictor of the probability to commit suicide, which coincides with the results presented by Pham and collaborators [22], where suicidal thoughts were predicted by emotional abuse.

Suicide is still a major global public health worry, and its prevention is critical to guaranteeing that millions do not continue being affected by the loss of loved ones [34]. The intervention and prevention of suicidal behavior is always complex, and the knowledge of specific issues that can be behind suicidal thoughts and attempts allows direct intervention in these factors. In this case, knowing that traumatic experiences have a role in predicting suicidal behavior, and the greater impact of particular experiences, enables work on the trauma caused by these specific experiences, reducing their impact on suicidal thoughts and attempts.

Being a pioneer on the theme of trauma, mental health, and suicidal behavior in the Community of Portuguese-Speaking Countries is a great advantage of this study. As far as we know, there is no investigation that assesses the variables in these populations. Our results clarify and emphasize the impact of traumatic experiences on mental health and its influence on suicidal behavior, contributing to better comprehension of these topics to support future interventions and highlighting the necessity of reducing the risk of exposure to traumatic events.

Our study shows important differences in the variables under study between the countries of residence. As stated earlier, the similarities between these countries should not outweigh the differences. One reason that may explain why war was reported with greater impact by ACPOL participants is the fact that these countries still live in situations of war and conflict, unlike Portugal and Brazil, where war is no longer present. On the other hand, Brazil is a country with less security and more acts of violence, which may justify the higher incidence of psychological and sexual abuse, as well as assaults and witnessed deaths.

Because it is a country with a lower human development index, Brazil’s investments and mental health policies may justify poor access to mental health care, leading to the worst levels of mental health found in our study. Furthermore, the reality of each country, including how its demographic and cultural differences influence perceptions about mental health and the existence of taboos around suicide, may explain a higher self-assessed probability of committing suicide reported by Portuguese participants, where more and more attention has been given to suicide in the past years.

However, the present study also presents some limitations, namely the questionnaire’s accessibility, since it was disseminated online and some ACPOL still lack technological resources, leading to a reduced number of participants from these countries. Additionally, the sample—owing to its higher educational level—was not representative of the population as a whole. Furthermore, the participant age at the time of trauma and the number of traumatic events were not considered, and these issues may have had some influence on psychological trauma. Also, the self-assessed probability of committing suicide was measured using only one item from de SBQ-R, making the results less strong. Future studies should address these limitations by carrying out investigations with more participants from ACPOL and analyzing the traumatic experiences in greater detail, taking into consideration the number of traumatic experiences, the age at which the trauma was experienced, and how much time has passed since that experience.

## 5. Conclusions

This study allowed us to identify the traumatic experiences most commonly experienced by the population of Portuguese Language Countries, and look at how those traumatic experiences overall contributed to the explanation of mental health levels and self-assessed probability to commit suicide, with greater contributions from psychological abuse, war, and witness to death.

These findings reinforce the importance and need to implement intervention strategies aimed at alleviating trauma, as well as campaigns to prevent suicidal behavior and promote the psychological well-being of populations. Considering the high incidence of violence and traumatic experiences in these countries, when implementing mental health policies and suicide prevention campaigns, the governments and policy makers of Portuguese Language Countries must consider the characteristics of their population and meet their needs. This study contributes to a better understanding of those traumatic experiences, and the impact they have on the population under study.

Given the important and delicate global issue of suicide, we hope that our results will encourage future research to explore in more detail the issues surrounding suicidal behavior in diverse populations in an effort to reduce the rate of suicidal thoughts and attempts.

## Figures and Tables

**Table 1:** Sociodemographic characteristics (Mean = 41.76; SD = 14.185).

Variable	Categories	*n*	%
Country of residence	Portugal	296	29.4
	Brazil	409	40.7
	ACPOL	301	29.9
Gender	Man	424	42.1
	Woman	576	57.3
	Other	6	0.6
Sexual orientation	Heterosexual	880	87.5
	Bisexual	66	6.5
	Gay/lesbian	60	6
Ethnicity/race	White/European	502	49.9
	African/black	253	25.1
	Mixed race	251	24.9
Professional situation	Employed	608	60.4
	Unemployed	47	4.7
	Student	143	14.2
	Student worker	82	8.1
	Self-employed	65	6.4
	Retired	52	5.2
	Sick leave	3	0.3
	Volunteering/community service	6	0.6
Educational attainment	Up to 12 years of schooling	100	9.9
	University—bachelor’s degree	249	24.7
	University—master’s degree	339	33.7
	University—doctorate	318	31.6
Socioeconomic status	Low	35	3.5
	Medium–low	109	10.8
	Medium	584	58.0
	Medium–high	227	22.5
	High	51	5.1
Place of residence	Small rural area	71	7.0
	Large rural area	27	2.7
	Small urban area	345	34.3
	Large urban area	563	56.0
Marital status	Single without dating	221	21.9
	Single with dating	169	16.8
	Married to same sex	14	1.4
	Married to different sex	372	37.0
	De facto union to same sex	12	1.2
	De facto union to different sex	134	13.3
	Separated or divorced same sex	11	1.1
	Separated or divorced different sex	60	5.9
	Widow of a different sex	13	1.3
Housing circumstances	Lives alone	172	17.1
	Lives with his/her partner	146	14.5
	Lives with his/her husband/wife	325	32.3
	Lives with his/her children	73	7.2
	Lives with his/her parents	169	16.8
	Live with his/her friends	47	4.7
	Another family constellation	74	7.3
Religion	Yes	676	67.2
	No	330	32.8

**Table 2:** Traumatic experiences by country of residence.

Experience	Country	0 Absence	1 Mild Intensity	2 Moderate Intensity	3 Severe Intensity	*χ^2^*	*p*
War	PT	26.1%	1.4%	1.2%	0.5%	52.418 (6)	0.000 **
	BR	39.5%	0.6%	0.6%	0.2%		
	ACPOL	23.8%	3.0%	1.8%	1.2%		
Accident	PT	20.6%	4.8%	2.4%	1.5%	2.708 (6)	0.845
	BR	29.5%	6.3%	3%	2%		
	ACPOL	22.6%	3.8%	1.9%	1.5%		
Disaster	PT	23.9%	3.2%	1%	1%	16.220 (6)	0.013
	BR	36.5%	2.5%	1.7%	0.2%		
	ACPOL	26.3%	1.8%	1%	0.7%		
Illness	PT	24.6%	1.2%	1.5%	2%	7.340 (6)	0.291
	BR	33.6%	2.4%	2.4%	2.1%		
	ACPOL	25.1%	2.2%	1.8%	0.9%		
Physical abuse	PT	22.7%	3.7%	1.8%	1.1%	6.600 (6)	0.359
	BR	31.3%	4.6%	2.6%	2.2%		
	ACPOL	22.3%	5%	1.3%	1.3%		
Psychological abuse	PT	18.3%	5.4%	2.5%	2.9%	24.059 (6)	0.001
	BR	22.2%	9.1%	4.6%	5%		
	ACPOL	20.5%	6.3%	2.1%	1.1%		
Sexual abuse	PT	26.2%	1.6%	0.3%	1.3%	28.952 (6)	0.000 **
	BR	31.7%	5%	1.8%	2%		
	ACPOL	26.8%	1.7%	1.1%	0.4%		
Assault	PT	22%	4.8%	1.3%	1%	85.356 (6)	0.000 **
	BR	17.3%	13.5%	7.1%	3.1%		
	ACPOL	17.3%	8.4%	2.2%	2%		
Death of a family member	PT	22.1%	2.4%	1.9%	2.9%	14.032 (6)	0.029 *
	BR	28.2%	4.5%	2.8%	5.2%		
	ACPOL	18.8%	4.7%	2.8%	3.5%		
Witness of death	PT	21.3%	4%	1.8%	2.3%	8.824 (6)	0.184
	BR	27.9%	5.6%	3.7%	3.6%		
	ACPOL	18.5%	5%	2.6%	3.6%		

* < 0.05; ** < 0.001; Note: PT—Portugal; BR—Brazil; ACPOL—African Countries with Portuguese as an Official Language.

**Table 3:** Differences in mental health symptoms by country of residence.

Symptom	Country	*M*	*SD*	*F(df)*	*p*
Somatization	PT	0.51	0.55	4.543 (2; 988)	0.011 *
	BR	0.64	0.69		
	ACPOL	0.52	0.57		
	Total	0.56	0.62		
Depression	PT	0.84	0.75	9.716 (2; 977)	0.000 **
	BR	1.04	0.82		
	ACPOL	0.79	0.73		
	Total	0.91	0.78		
Anxiety	PT	0.92	0.70	17.688 (2; 974)	0.000 **
	BR	1.12	0.86		
	ACPOL	0.77	0.69		
	Total	0.96	0.78		
Overall symptoms	PT	0.76	0.58	11.101 (2; 991)	0.000 **
	BR	0.93	0.71		
	ACPOL	0.71	0.63		
	Total	0.81	0.66		

* < 0.05; ** < 0.001; Note: PT—Portugal; BR—Brazil; ACPOL—African Countries with Portuguese as an Official Language.

**Table 4:** Differences in suicidal behavior by country of residence.

Variable	Country	*M*	*SD*	*F(df)*	*p*
SBQ 1—Lifetime suicidal thoughts	PT	1.48	0.60	11.441 (2; 945)	0.000 **
	BR	1.56	0.66		
	ACPOL	1.33	0.55		
	Total	1.46	0.61		
SBQ 2—Lifetime suicidal attempts	PT	1.17	0.58	0.451 (2; 989)	0.637
	BR	1.20	0.62		
	ACPOL	1.16	0.55		
	Total	1.18	0.59		
SBQ 3—Suicidal thoughts in the last year	PT	1.33	0.86	7.112 (2; 987)	0.001
	BR	1.44	0.92		
	ACPOL	1.20	0.63		
	Total	1.33	0.83		
SBQ 4—Suicidal attempts in the last year	PT	1.06	0.38	1.913 (2; 986)	0.148 *
	BR	1.03	0.23		
	ACPOL	1.08	0.42		
	Total	1.05	0.39		
SBQ 5—Probability of committing suicide	PT	1.44	0.97	5.525 (2; 981)	0.004 *
	BR	1.38	0.99		
	ACPOL	1.20	0.71		
	Total	1.35	0.91		

* < 0.05; ** < 0.001; Note: PT—Portugal; BR—Brazil; ACPOL—African Countries with Portuguese as an Official Language.

**Table 5:** Hierarchical linear regression analysis predicting mental health.

Variable	Model 1			Model 2			Model 3		
	*B*	*SE B*	*β*	*B*	*SE B*	*β*	*B*	*SE B*	*β*
Age	−0.007	0.002	−0.155 **	−0.007	0.002	−0.157 **	−0.009	0.002	−0.207 **
Gender	0.184	0.042	0.142 **	0.189	0.043	0.146 **	0.126	0.041	0.098 **
Sexual orientation	0.113	0.040	0.091 **	0.116	0.041	0.093 **	0.018	0.040	0.014 *
Socioeconomic status	−0.158	0.027	−0.194 **	−0.156	0.027	−0.192 **	−0.141	0.026	−0.173 **
Country of residence				0.014	0.028	0.016 *	0.008	0.027	0.010 *
War							0.042	0.037	0.038 *
Accident							0.011	0.025	0.014 *
Disaster							−0.055	0.034	−0.052 *
Illness							0.072	0.025	0.090 **
Physical abuse							0.013	0.035	0.015 *
Psychological abuse							0.161	0.028	0.241 **
Sexual abuse							0.057	0.033	0.060 **
Assault							0.001	0.024	0.001 *
Death of a family member							0.009	0.020	0.014 *
Witness of death							0.084	0.022	0.129 **
*R* ^2^			0.116			0.116			0.234
F			28.798 **			23.064 **			17.699 **

* *p* < 0.05; ** *p* < 0.001.

**Table 6:** Hierarchical linear regression analysis predicting the probability of committing suicide.

Variable	Model 1			Model 2			Model 3		
	*B*	*SE B*	*β*	*B*	*SE B*	*β*	*B*	*SE B*	*β*
Age	−0.001	0.002	−0.018 *	−0.001	0.002	−0.012 *	−0.003	0.002	−0.048 *
Gender	0.075	0.062	0.041 *	0.042	0.063	0.023 *	−0.020	0.064	−0.011 *
Sexual orientation	0.374	0.059	0.213 **	0.355	0.060	0.203 **	0.252	0.061	0.144 **
Socioeconomic status	−0.080	0.040	−0.069 **	−0.091	0.040	−0.079 **	−0.077	0.039	−0.67 *
Country of residence				−0.094	0.041	−0.078 **	−0.099	0.041	−0.082 **
War							0.018	0.056	0.011 *
Accident							0.082	0.038	0.074 **
Disaster							−0.072	0.052	−0.048 *
Illness							0.060	0.038	0.053 *
Physical abuse							−0.018	0.053	−0.015 *
Psychological abuse							0.142	0.043	0.150 **
Sexual abuse							0.092	0.051	0.068 *
Assault							0.045	0.036	0.043 *
Death of a family member							−0.004	0.031	−0.004 *
Witness of death							0.045	0.034	0.049 *
*R* ^2^			0.055			0.060			0.116
F			12.617 **			11.179 **			7.500 **

* *p* < 0.05; ** *p* < 0.001.

## Data Availability

Data available upon request.

## Supplementary Materials

The following supporting information can be downloaded at: https://www.mdpi.com/article/10.3390/bs12040102/s1, Table S1: Incidence of traumatic experiences; Table S2: Results for mental health symptoms; Table S3: Incidence of suicidal behavior; Table S4: Correlation values among mental health symptoms and traumatic experiences; Table S5: Correlation values among suicidal behavior and traumatic experiences.
