# Supplementary material for: The Impact of Life Trauma on Mental Health and Suicidal Behavior: A Study from Portuguese Language Countries
Source: Behav Sci (Basel). 2022 Apr 10;12(4):102. doi: 10.3390/bs12040102 (PMC9031010; doi:10.3390/bs12040102)
Supplement: Supplementary file 1 [file behavsci-12-00102-s001.zip › behavsci-1630180-supplementary.pdf]

Table S1. Incidence of traumatic experiences.

Experience	Trauma intensity	n	%
War	0	890	89.4
	1	51	5.1
	2	36	3.6
	3	19	1.9
Accident	0	731	72.7
	1	150	14.9
	2	75	7.4
	3	50	5
Disaster	0	872	86.7
	1	77	7.6
	2	38	3.8
	3	19	1.9
Illness	0	838	83.3
	1	60	5.9
	2	58	5.8
	3	50	5
Physical abuse	0	767	76.2
	1	135	13.4
	2	58	5.8
	3	46	4.6
Psychological abuse	0	615	61.1
	1	208	20.7
	2	93	9.2
	3	90	9
Sexual abuse	0	852	84.7
	1	83	8.3
	2	32	3.2
	3	39	3.8
Assault	0	569	56.6
	1	268	26.6
	2	107	10.6
	3	62	6.2
Death of a family member	0	694	69
	1	118	11.7
	2	76	7.6
	3	118	11.7
Witness of death	0	681	67.7
	1	146	14.5
	2	82	8.2
	3	97	9.6

Table S2. Results for mental health symptoms.

Symptom	Frequency	n	%	Mean	S.D.
Anxiety	Never	572	56.9	0.96	0.779
	Few times	318	31.6		
	Sometimes	100	9.9		
	Often	13	1.3		
	Many times	3	0.3		
Depression	Never	590	58.7	0.91	0.781
	Few times	303	30.1		
	Sometimes	90	8.9		

	Often	19	1.9		
	Many times	4	0.4		
Somatization	Never	774	76.9	0.56	0.622
	Few times	194	19.3		
	Sometimes	32	3.2		
	Often	6	0.6		
	Many times	0	0		

Table S3. Incidence of suicidal behavior.

**Variable**	**Frequency**	**n**	**%**
SBQ 1 - Lifetime suicidal thoughts	Never	604	60
	Only a passing thought	337	33.5
	I had a plan to commit suicide, at least once but I never accomplished	65	6.5
	anything	0	0
	I had a plan to commit suicide and I really wanted to die		
SBQ 2 - Lifetime suicidal attempts	Never	905	89.9
	Just a fleeting attempt	52	5.2
	I tried to commit suicide, but I didn't really want to die	24	2.4
	I tried to commit suicide and I really wanted to die	25	2.5
SBQ 3 - Suicidal thoughts in the last year	Never	821	81.6
	Rarely (once)	103	10.2
	Sometimes (twice)	35	3.5
	Several times (three or four times)	29	2.9
	Often (five or more times)	18	1.8
SBQ 4 - Suicidal attempts in the last year	Never	973	96.8
	Rarely (once)	17	1.7
	Sometimes (twice)	10	1
	Several times (three or four times)	3	0.3
	Often (five or more times)	2	0.2
SBQ 5 - Probability of committing suicide (1 = not likely - 7 = very likely)	1	813	80.8
	2	121	12
	3	32	3.2
	4	17	1.7
	5	11	1.1
	6	6	0.6
	7	6	0.6

Table S4. Correlation values among mental health symptoms and traumatic experiences.

	1	2	3	4	5	6	7	8	9	10	11	12	13
1- ANX	-												
2- DEP	0.776**	-											
3- SOM	0.682**	0.592**	-										
4- T1	-0.040	-0.001	0.010	-									
5- T2	0.022	0.036	0.078*	0.222**	-								
6- T3	0.051	0.022	0.054	0.177**	0.217**	-							
7- T4	0.101**	0.059	0.121**	0.069*	0.155**	0.134**	-						
8- T5	0.228**	0.228**	0.195**	0.061	0.181**	0.175**	0.092**	-					
9- T6	0.331**	0.309**	0.233**	-0.013	0.117**	0.160**	0.098**	0.658**	-				
10- T7	0.229**	0.189**	0.209**	-0.017	0.091**	0.091**	0.068*	0.469**	0.486**	-			
11- T8	0.113**	0.076*	0.098**	0.069*	0.185**	0.174**	0.168**	0.187**	0.206**	0.120**	-		
12- T9	0.070*	0.067*	0.043	0.167**	0.151**	0.117**	0.068*	0.175**	0.121**	0.104**	0.181**	-	
13- T10	0.102*	0.102*	0.116**	0.234**	0.216**	0.180**	0.173**	0.151**	0.088**	0.090**	0.252**	0.315**	-

*p<0.05;

**p<0.01

Table S5. Correlation values among suicidal behavior and traumatic experiences.

	1	2	3	4	5	6	7	8	9	10	11	12	13	14	15
1- SBQ1	-														
2- SBQ2	0.413**	-													
3- SBQ3	0.526**	0.342**	-												
4- SBQ4	0.170**	0.448**	0.386**	-											
5- SBQ5	0.403**	0.401**	0.566**	0.456**	-										
6- T1	-0.042	-0.048	-0.013	0.012	-0.006	-									
7- T2	0.012	0.034	0.007	0.071*	0.095**	0.222**	-								
8- T3	0.040	0.042	0.002	0.029	0.035	0.177**	0.217**	-							
9- T4	0.068*	0.104**	0.046	0.134**	0.095**	0.069*	0.155**	0.134**	-						
10- T5	0.162**	0.191**	0.183**	0.118**	0.155**	0.061	0.181**	0.175**	0.092**	-					
11- T6	0.289**	0.238**	0.242**	0.223**	-0.013	0.117**	0.160**	0.098**	0.658**		-				
12- T7	0.201**	0.225**	0.153**	0.086**	0.175**	-0.017	0.091**	0.091**	0.068*	0.469**	0.486	-			

13- T8	0.081*	0.059	0.073*	0.030	0.115**	0.069*	0.185**	0.174**	0.168**	0.187**	0.206**	0.120**	-		
14- T9	-0.006	0.060	0.031	0.088*	0.031	0.167**	0.151**	0.117**	0.068*	0.175**	0.121**	0.104**	0.181**	-	
15- T10	0.099**	0.046	0.069*	0.067*	0.072*	0.234**	0.216**	0.180**	0.173**	0.151**	0.088**	0.090**	0.252**	0.315**	-

*p<0.05

**p<0.
